# Pharmacopœa Borussica

**Published:** 1800-01

**Authors:** 


### CRITICAL RETROSPECT OF MEDICAL AND PHYSICAL LITERATURE.

[ FOREIGN AND DOMESTIC. ]

### PHARMACY.

*Pharmacopœa Borussica,* 1799, 223 pp. quarto, *Berlin,* Decker.

The laſt edition of the Pruſſian Diſpenſatory appeared in 1781, and has long maintained a diſtinguiſhed place among the foreign Pharmacopœas; but the rapid progreſs of Chemiſtry and other auxiliary ſciences, together with various attempts made by individuals to compoſe ſyſtems of pharmacy, more conſiſtent with the preſent ſtate of medical knowledge, induced the Pruſſian College to publiſh a new and corrected Diſpenſatory. To accompliſh this deſirable purpoſe, the preſent miniſter of the literary department, Count DE SCHULENBURG-KEHNERT, appointed a committee of the moſt able and experienced phyſicians in the kingdom, who have honourably acquitted themſelves of their charge.

Although the authors of this work have carefully expunged many of the old and abſurd compoſitions of drugs, and ſubſtituted others more ſimple and leſs liable to decompoſition, yet, in our opinion, much remains to be done in this eſſential department of medical practice. To verify this aſſertion, we ſhall quote ſome of the compound remedies which ſtill conſiſt of ſix or eight different ingredients; for inſtance, the *aromatic water,* and the *ſtomachic elixir* of the Berlin College: the former is prepared by diſtilling eight ounces of ſage leaves, four ounces of roſemary, the ſame quantity of peppermint and lavender flowers, two ounces of fennel-ſeed, two ounces of cinnamon, four pounds of rectified ſpirit of wine, and a proportionate quantity of water. The latter is rather more complicated, and requires the following proceſs: Four ounces of ripe orange peel, two ounces of the unripe peel, a ſimilar quantity of cinnamon, and one ounce of aërated dry vegetable alkali, are firſt infuſed in four pounds of Malaga wine, and left for ſeveral days to digeſt in a warm place; after which the tincture is properly ſtrained, and then are added four ounces of bitter extract, two drachms of the oil of lemons, and two ounces of the anodyne liquor.—Inſtead of the former *pectoral elixir,* the authors recommend a ſolution of the purified extract of liquorice in ſix ounces of fenel-water, with the addition of one ounce of laudanum, and ſix ounces of ſpirit of ſal ammoniac. The directions here given for preparing the tooth-powder, the tincture of aſafœtida, the extract of quaſſia, and ſeveral other other medicines, appear to us fufceptible of improvement, or at leaft fimplification of the procefs. Upon the whole, however, the prefent work deferves the preference over moft other foreign Pharmacopœas, and will doubtlefs be ftill farther improved in fubfequent editions. W.

